# Long-term assessment of quality of life in the Intergroup Exemestane Study: 5 years post-randomisation

**DOI:** 10.1038/bjc.2012.43

**Published:** 2012-02-21

**Authors:** L J Fallowfield, L S Kilburn, C Langridge, C F Snowdon, J M Bliss, R C Coombes

**Affiliations:** 1 Sussex Health Outcomes Research & Education in Cancer (SHORE-C), Brighton & Sussex Medical School, University of Sussex BN1 9RX, Brighton, UK; 2 Clinical Trials & Statistics Unit (ICR-CTSU), Division of Clinical Studies, The Institute of Cancer Research, Sutton, UK; 3 Division of Cancer, Imperial College London, London, UK

**Keywords:** aromatase inhibitor, breast cancer, exemestane, oestrogen receptor, quality of life

## Abstract

**Background:**

The Intergroup Exemestane Study (IES) (ISRCTN11883920) demonstrated improved survival for postmenopausal women with ER-positive/unknown primary breast cancer who switched to exemestane after 2–3 years tamoxifen, compared with those continuing on tamoxifen to complete 5 years therapy. This was achieved without detriment to on-treatment quality-of-life (QoL). We report on- and post-treatment QoL impact in IES.

**Methods:**

A total of 582 patients from 8 countries participated in the QoL substudy. Functional Assessment of Cancer Therapy–Breast (FACT-B) and endocrine symptom subscale (ES) were completed at baseline, 3, 6, 9, 12, 18, 24, 30, 36, 48 and 60 months. The primary endpoint was FACT-B Trial Outcome Index (TOI); secondary endpoints included severity of individual endocrine symptoms.

**Results:**

Both the groups showed gradual improvement in overall QoL and lessening of total endocrine symptoms post treatment compared with baseline (*P*<0.002). There was no evidence of any between-group differences in TOI. Vasomotor complaints remained high on treatment. Vaginal discharge was more frequent (*P*<0.01) with tamoxifen up to 24 months from baseline. In both the groups, post-treatment libido did not recover to baseline levels.

**Conclusion:**

Clinical benefits of switching to exemestane are accompanied by good overall QoL. Although some symptoms persist, the majority of endocrine symptoms improve after treatment completion.

Following the publication of significant results from several large phase III, randomised, double-blind controlled trials of aromatase inhibitors (AIs) in postmenopausal women with hormone receptor positive primary breast cancer, adjuvant AI use has become standard in this patient population. One such trial, the Intergroup Exemestane Study (IES), randomised postmenopausal women with ER positive or ER unknown primary breast cancer, disease-free after 2–3 years of tamoxifen treatment, to either switch to exemestane or to continue tamoxifen until completion of 5 years endocrine treatment. Mature data from the IES, with a median follow-up of 91 months, demonstrated that switching to exemestane improves both disease-free and overall survival with serious side-effects rare in both the groups (Bliss *et al*, 2011).

The first results from the IES Quality-of-Life (QoL) sub-protocol 24 months post randomisation demonstrated that patients reported the switch neither increased nor decreased endocrine symptoms present following 2–3 years of tamoxifen, nor initiated significant reports of new ones (Fallowfield *et al*, 2006). Thus, the clinical benefits observed in IES seem to be achieved without a detrimental impact on overall QoL.

Although patient reported QoL has been documented during study treatment, few adjuvant hormonal trials to date have reported long-term QoL results post study treatment completion. Here, we report QoL in the IES through to 60 months by randomised treatment; equivalent to 7.5 years after start of endocrine treatment and 2.5 years after completion of endocrine treatment, allowing exploration of persistence or resolution of side-effects post-treatment and relationship with trial treatment.

## Methods

Details of the eligibility, assessments and methods of the QoL substudy have been provided previously (Fallowfield *et al*, 2006). Briefly, women from a subset of 91 IES centres in 8 countries participating in the main IES were eligible to join the QoL substudy. All were postmenopausal women with primary breast cancer that was either ER-positive or ER-unknown who had completed surgery, plus radiotherapy and/or chemotherapy according to local practice, and had received 2–3 years of adjuvant tamoxifen.

Assessment of QoL was made with the Functional Assessment of Cancer Therapy – Breast (FACT-B) (Brady *et al*, 1997) and Endocrine Subscale (ES) (Fallowfield *et al*, 1999). Questionnaires were administered at baseline (before randomisation) and before follow-up clinics at 3, 6, 9, 12, 18, 24, 30, 36, 48 and 60 months post randomisation. Women were asked to complete the questionnaires without assistance from clinical staff. In the UK all follow-up questionnaires were administered by post from the CRUK Psychosocial Oncology Group Co-ordinating Centre. In all other countries, patients were requested to complete the questionnaires before their clinic visits. All questionnaires were collated by the co-ordinating centre staffs who were blinded to treatment allocation.

The primary endpoint of the QoL study was the trial outcome index (TOI); a summation of the physical, functional and breast cancer concerns subscales. Higher TOI scores are associated with better QoL, whereas a difference of 5 in the TOI is considered to be the clinically relevant minimally important difference (Webster *et al*, 2003). Secondary outcomes were the overall ES scores, total combined FACT-B and ES scores and the severity of individual symptoms established *a priori* as being of primary importance. Endocrine Subscale items are negatively framed; thus, scores were reversed for analysis so that high scores equate to a good QoL.

### Statistical methods

To detect a difference in change from baseline and at each follow-up of 5 in the TOI between the two treatment groups required 235 patients per group for the trial to have 95% power and a significance level of 5%.

The primary analysis was the mean change from baseline in the TOI between the two treatment groups (exemestane-tamoxifen; thus positive mean change favours exemestane) at each time point using two-sample (between group) *t*-tests. Within group mean changes were analysed using paired *t*-tests (positive mean change indicates improvement in QoL from baseline). Mean change from baseline in the ES and FACT-B+ES scores between groups were also analysed using *t*-tests. To take into account multiple time-points, repeated measures modelling using generalised estimating equations (GEE) was performed on TOI, ES, FACT-B+ES scores adjusting for allocated treatment, baseline QoL score, time from randomisation and the *a priori* specified factors of ER status (negative *vs* positive), nodal status (node negative, 1–3 positive nodes, or ⩾4 positive nodes), chemotherapy use (yes *vs* no), and HRT use before random assignment (yes *vs* no). Coefficients >0 favour exemestane. GEE models not adjusting for the pre-specified factors gave similar results (data not shown). Models used unstructured correlation or exchangeable correlation where models did not converge using the unstructured matrix. Polynomial effects and interactions were explored but did not improve the fit of the models.

Prevalence of severe cases of individual ES symptoms was calculated for each time-point, for each treatment group and analysed using *χ*^2^ tests. Severity is defined as ‘quite a bit’ or ‘very much’ on the QoL questionnaires. Forest plots representing odds ratios and 95% confidence intervals (CIs) for the treatment effect adjusting for baseline QoL score and time from randomisation, calculated using GEE, are presented for individual items.

All analyses were conducted on a QoL data snapshot processed by 20 April 2007 and a main clinical study data snapshot of 7 December 2009. Results are presented for all patients randomised into the study with a valid TOI and ES score at baseline and comparisons are made by randomised treatment. Intention to treat analyses was performed throughout. Sensitivity analyses by treatment received, excluding main study protocol violators (*n*=23) and including only countries where patients demonstrated good compliance, did not significantly alter the conclusions and thus these data are not shown here. To account for multiple testing *P*<0.01 was deemed statistically significant for all endpoints.

Analysis of data was conducted by statisticians at the Clinical Trials & Statistics Unit (ICR-CTSU), The Institute of Cancer Research, Sutton, UK. At no time before the analysis or interpretation of results did the sponsor have access to the QoL database.

## Results

### Demographics

A total of 582 patients from the US (*n*=280), UK (173), Spain (50), Argentina (40), Italy (25), Australia (8), Netherlands (4) and New Zealand (2) participated in the QoL substudy. There were no significant differences between groups at baseline in age, tumour size, nodal status, ER or PgR status, histological grade, surgery, primary localisation, prior use of HRT, histological type, chemotherapy, radiotherapy or country. Median randomised treatment duration for QoL patients was 30.7 months (IQR: 24.4, 34.9) with 127 (21.8%) discontinuing randomised treatment early.

### Questionnaire completion

**Figure 1:**
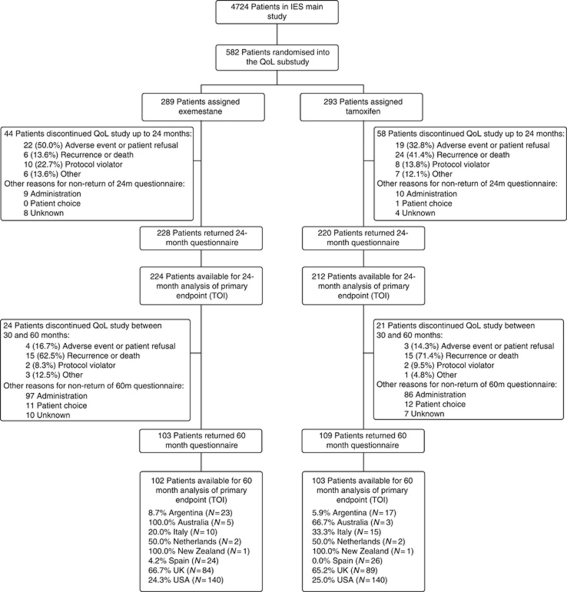
CONSORT flow diagram.

Overall, questionnaire completion was good with 4717 questionnaires returned out of 5581 sent (84.5%) during the study. Main reasons for discontinuation were recurrence/death and adverse events (Figure 1). However, after expected study treatment cessation between 30–36 months, questionnaire compliance decreased with noticeable differences between countries. For this reason, an analysis of data from only those countries with ⩾50% compliance at 60 months (Australia, Netherlands, New Zealand and the United Kingdom) was conducted. As this sensitivity analysis did not alter the conclusions of the main analysis, it is not shown here.

### Primary endpoint – TOI

**Figure 2:**
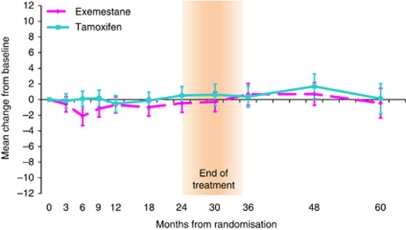
Mean change from baseline TOI scores within treatment groups. Error bars denote 95% CIs. Between treatment group: positive mean change in QoL favours exemestane. Within treatment group: positive mean change in QoL indicates improvement from baseline. Mean (s.d.) baseline TOI scores are exemestane=73.1 (10.9); tamoxifen=72.1 (11.5).

There was no evidence of a difference in change from baseline TOI score between the treatment groups at 24 months (mean change: −0.98 points, 95% CI: −2.62, 0.67; *P*=0.24). Post treatment, this continued with no evidence of a difference between treatments at 30–60 months (mean change at 60m: -0.57 points, 95% CI: −3.30, 2.16; *P*=0.68). Similarly, the GEE model for TOI showed no evidence to suggest a difference in the mean TOI score over time between the treatment groups (adjusted treatment coefficient=−0.54, 95% CI: −1.60, 0.52; *P*=0.32). In addition, there was no evidence of a difference in change from baseline TOI score within the treatment groups at all time points on and post-treatment completion except for exemestane at 6 months (mean change: −2.10, 95% CI: −3.32, −0.89; *P*=0.001) (Figure 2). This change, likely due to chance, was noted previously (Fallowfield *et al*, 2006) and is not considered clinically meaningful.

### Secondary endpoints

#### Endocrine subscale score

**Figure 3:**
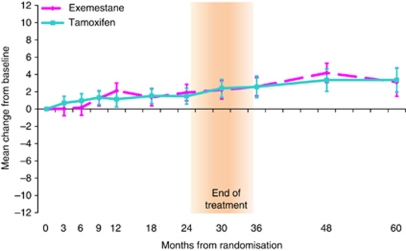
Mean change from baseline ES scores within treatment groups. Error bars denote 95% CIs. Between treatment group: positive mean change in QoL favours exemestane. Within treatment group: positive mean change in QoL indicates improvement from baseline. Mean (s.d.) baseline ES scores are exemestane=57.0 (9.1); tamoxifen=56.7 (9.8).

Similar to TOI, there was no evidence of a difference between the treatment groups in the change in ES score from baseline at any time point (mean change at 24 months: 0.42, 95% CI: −0.91, 1.74; *P*=0.54 and mean change at 60 months: −0.25, 95% CI: −2.40, 1.89; *P*=0.82). The GEE model also showed no evidence of a difference between the treatment groups over time (adjusted treatment coefficient=0.01, 95% CI: −0.85, 0.88; *P*=0.98). Within both treatment groups patients reported a lessening of endocrine symptoms over time on treatment and during the post-trial treatment period (Figure 3).

#### FACT-B+ES score

**Figure 4:**
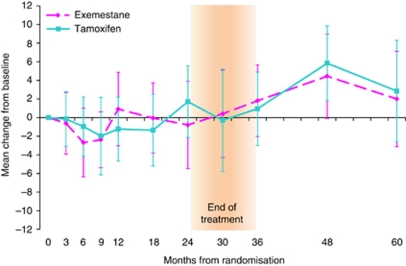
Mean change from baseline FACT B+ES scores within treatment groups Error bars denote 95% CIs. Between treatment group: positive mean change in QoL favours exemestane. Within treatment group: positive mean change in QoL indicates improvement from baseline. Mean (s.d.) baseline TOI scores are exemestane=180.8 (23.5); tamoxifen=178.9 (24.7).

The addition of FACT-B to ES did not significantly alter the results. There was no evidence of a difference between the treatment groups in the change in FACT-B+ES score from baseline at any time point (mean change at 24 months: −2.72, 95% CI: −6.42, 0.99; *P*=0.15 and mean change at 60 months: −1.14, 95% CI: −7.54, 5.26; *P*=0.73). The GEE model also showed no evidence of a difference between the treatment groups over time (adjusted treatment coefficient=−1.02, 95% CI: −3.33, 1.29; *P*=0.39). Within both treatment groups there was no evidence of a difference in QoL from baseline at all time points, except at 6 months there was worse QoL for exemestane (mean change −3.10, 95% CI: −5.35, −0.85; *P*=0.007) and at 48 months a better QoL for tamoxifen (mean change 4.99, 95% CI: 2.04, 7.93; *P*=0.001). As for TOI, these changes are likely due to chance and are not clinically relevant (Figure 4).

### Severity of individual endocrine symptoms

#### Vasomotor symptoms

**Figure 5:**
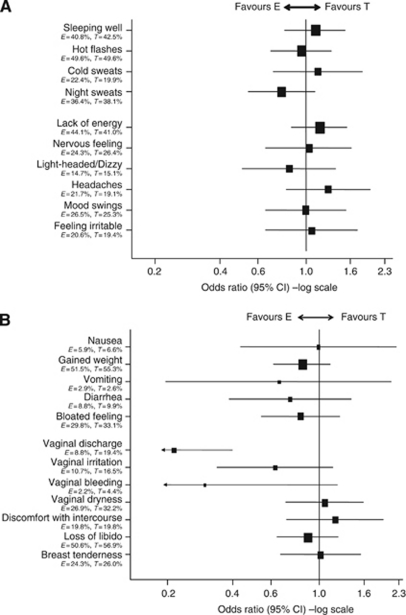
(**A**) Vasomotor and neuropsychological symptoms. (**B**) Gastrointestinal and gynaecological symptoms.

Severe vasomotor complaints, especially hot flushes and night sweats, remained problematic for both the groups with >19% reporting at least one of these symptoms at some point during the study (Figure 5A). Post-treatment completion the percentage of women reporting severe hot flushes, night sweats and cold sweats decreased (Supplementary appendix: Figures A2, A3, A6); however, patients continued to report poor sleep beyond treatment completion (Supplementary appendix: Figure A1).

#### Neuropsychological symptoms

There was no evidence of a difference between the treatment groups over time for any of the neuropsychological symptoms. Lack of energy was the most commonly reported symptom with >41% patients identifying this as severe in both the groups (Figure 5A). Post-treatment completion the percentage of women reporting severe lack of energy remained at similar levels as on-treatment along with light-headedness/dizziness, headaches, mood swings and irritable feeling, although these were less common on-treatment (Web appendix: Figures A4, A14, A15, A8, A16). Prevalence of severe nervous feeling decreased post treatment (Supplementary appendix: Figure A7).

#### Gastrointestinal symptoms

Weight gain and feeling bloated were the most commonly reported gastrointestinal symptoms. Diarrhoea and nausea were reported less often, whereas vomiting was rarely reported. Again, there was no evidence of a difference between the treatment groups in the reporting of these symptoms over time (Figure 5B). Post treatment the percentage of women reporting severe weight gain and diarrhoea decreased, whereas bloated feeling continued at similar levels to those reported on-treatment. Severe nausea and vomiting continued to be rarely reported (Supplementary appendix: Figures A5, A9, A10, A17, A18).

#### Gynaecological symptoms

**Figure 6:**
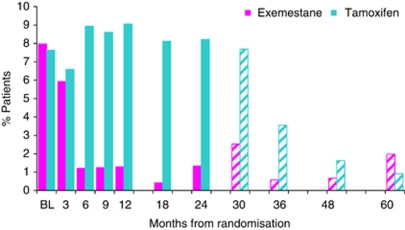
Reporting of severe vaginal discharge over time in the QoL study. Key: Solid bars indicate % patients reporting severe vaginal discharge on treatment; Hatched bars indicate % patients reporting severe vaginal discharge post treatment.

**Figure 7:**
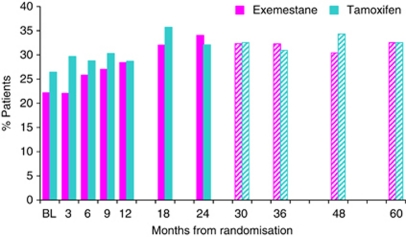
Reporting of severe loss of libido over time in the QoL study. Key: Solid bars indicate % patients reporting severe loss of libido on treatment; Hatched bars indicate % patients reporting severe loss of libido post treatment.

Reports of severe vaginal discharge were significantly higher (*P*<0.01) over time with tamoxifen (Figure 5B). Differences were seen between treatments at 6, 9, 12, 18 and 24 months, whereas post-study treatment completion there was no evidence of a difference between the two groups with reports in the tamoxifen group reducing dramatically (Figure 6). Libido never recovered to baseline levels for either group, even after cessation of treatment. At 60 months 27 out of 83 (32.5%) (22.6 to 43.7%) patients in the exemestane group and 27 out of 83 (32.5%) (22.6 to 43.7%) patients in the tamoxifen group reported a considerable loss of libido (Figure 7). Severe vaginal irritation and bleeding were rare at all time points, whereas reporting of severe vaginal dryness, discomfort with intercourse and breast tenderness post-treatment continued at similar levels to those reported on-treatment (Supplementary appendix: Figures A19, A20, A11, A12, A13).

## Discussion

There are very few long-term reports of QoL using patient-reported outcomes from standardised measures in women who have had hormone therapy for breast cancer for 5 years. The persistence of different endocrine symptoms, which although not life threatening can be detrimental to QoL, are largely unknown compared with acute short-term side-effects.

The IES on-treatment results are consistent with the findings from the other main AI trials with patient-reported outcomes in that AIs are well tolerated with good overall QoL (Whelan *et al*, 2005; Cella *et al*, 2006). The TEAM trial published self-reported neuropsychological outcomes and showed that neither tamoxifen nor exemestane increased the reports of cognitive failures compared with healthy controls (Schilder *et al*, 2011). However, little data are available on the post-treatment impact on QoL in these studies (Fallowfield, 2007). Therefore, IES is the only major study reported to date showing the decline in patient-reported symptoms following AI or tamoxifen treatment. It is important to note, however, that compliance of questionnaire completion post treatment was significantly reduced, particularly in countries where questionnaires were completed in clinics before appointments. Higher completion rates were achieved when questionnaires were sent out by post, directly to the patient, as was the case in UK, suggesting that this may be the method of choice for future studies.

The published reports of side-effects captured by physicians on case report forms tend to focus on any report of a symptom at any time point. Although this is a useful summary for safety analysis, this is not necessarily helpful clinically or to aid patient choice. Previous reports from the IES QoL substudy have shown the temporal relationship of symptom development. These have shown that endocrine-related symptoms tend to increase over the first 3 months of treatment and thereafter either stabilise or abate. In the current analysis through to 5 years post randomisation, the temporal pattern of decline in symptoms is captured. For the vast majority of women most of the vasomotor and gynaecological problems disappear completely. The notable exception is for severe loss of libido, which remains for >30% women irrespective of allocated treatment group. Sexual behaviour is complex but previous explanations for a loss of libido with hormone therapy in breast cancer have included mood-state changes. Examples include anxiety and depression or gynaecological symptoms such as vaginal dryness or discharge and irritation associated with dyspareunia (Cella and Fallowfield, 2008). In previous studies, it has been shown that women at increased risk of cancer taking part in chemoprevention trials and those in a bilateral prophylactic mastectomy study are as sexually active as healthy controls (Atkins and Fallowfield, 2007). The diagnosis of cancer does appear to diminish sexual activity compared with that in healthy postmenopausal women (Conde *et al*, 2005) as does increasing age (Addis *et al*, 2006; Atkins and Fallowfield, 2007). While on treatment, the gynaecological symptoms and lowered oestrogen levels might have created a loss of sexual desire that then never recovered again. Although a loss of libido is not life threatening, it can be quality of life threatening and affect relationships. Consideration might be given to offering patients interventions such as lubricants or couple counselling pro-actively during endocrine treatment so that a habitual lack of sexual activity due to dyspareunia or altered mood state is not allowed to develop into a chronic state. How willing healthcare professionals may be to monitor this, how willing patients might be to accept it and how effective an intervention it would be, needs testing in further trials.

The clinical benefits of switching to exemestane after 2–3 years tamoxifen are accompanied by good QoL up to 7–8 years post-initial endocrine therapy. Following treatment completion, the majority of endocrine symptoms improved; however, further research is needed to develop interventions to ameliorate the vasomotor and sexual symptoms associated with hormonal therapies in women treated for early breast cancer.
