# Supplementary material for: Long-term assessment of quality of life in the Intergroup Exemestane Study: 5 years post-randomisation
Source: Br J Cancer. 2012 Feb 21;106(6):1062–7. doi: 10.1038/bjc.2012.43 (PMC3304414; doi:10.1038/bjc.2012.43)
Supplement: Supplementary Appendix [file bjc201243x1.doc]

**Appendix**

Investigators participating into the Quality of Life Sub-protocol: S Vukelja, Tyler Cancer Center, Tyler, USA (27); R Bastús, Hospiatl Mutua de Terrassa, Terrassa, Spain (24); T Marsland, Florida Oncology Association, Orange park, USA (23); CP Hinton, Princess Royal Hospital, Telford, UK (22); S Jones, Iowa Cancer Center, Cedar Rapids, USA (21); A Cohn, RMCC Colorado Springs, Colorado Springs, USA (18); C Coombes, Charing Cross Hospital, London, UK (18); S Anthony, Cancer Care Northwest, Spokane, USA (16); A Balil, Hospital Arnau de Vilanova, Lleida, Spain (14); C Ghosh, Berkshire Hematology/Oncology, Pittsfield, USA (14); J Fox, Castle Hill Hospital, Cottingham, UK (14); J Jadeja, Hematology/Oncology Association, Jacksonville, USA (14); L Fein, Centro Oncologico de Rosario, Rosario, Argentina (14); T Anderson, Texas Oncology Physicians Association, Euless, USA (14); A Stewart, Christie Hospital, Manchester, UK (12); J Cantrell, Bruno Cancer Center, Birmingham, USA (12); MF Bruno, Hospital Alvarez, Buenos Aires, Argentina (12); S Chittor, Texas Cancer Center of Mesquite, Mesquite, USA (12); D Dodwell, R Knox, Harrogate District Hospital, Harrogate, UK (11); A Nejim, Airedale General Hospital, Keighley, UK (10); A Otsuka, Rocky Mountain Cancer Center, Thornton, USA (9); E Bajetta, Istituto Nazionale Tumori, Milan, Italy (9); K Mcintyre, Texas Oncology-Presbyterian, Dallas, USA (9); M Brunt, North Staffordshire Royal Infirmary, Stoke-on-Trent, UK (8); SJ Cawthorn, Frenchay Hospital, Bristol, UK (8); A Otsuka, RMCC Lakewood, Lakewood, USA (7); EA Gardiol, Centro de Mastologia de Rosario, Rosario, Argentina (7); K Horgan, D Dodwell, Leeds General Infirmary, Leeds, UK (7); M Savin, North Texas Regional Cancer Center, Plano, USA (7); R Coleman, Weston Park Hospital, Sheffield, UK (7); S Goodman, Yeovil Hospital, Yeovil, UK (7); T Joannides, RCF Leonard, Singleton Hospital, Swansea, UK (7); A Makris, Mount Vernon Hospital, London, UK (6); D Gruenberg, Northwest Cancer Specialists, Portland, USA (6); D Loesch, Oncology and Hematology Assoc Inc, Indianapolis, USA (6); D Loesch, US Oncology, Indianapolis, USA (6); E Galindo; BJ Marek, South Texas Cancer Center, Mcallen, USA (6); H Zimbler, Texas Oncology Physicians Association, Dallas, USA (6); J Chirgwin, Box Hill Hospital, Melbourne, Australia (6); J Lizon Giner, Hospital Universitari Sant Joan D'Alcant, Alicante, Spain (6); M Tonato, Policlinico Monteluce, Perugia, Italy (6); MR Modiano, ACRC, Tucson, USA (6); T George, West Texas Cancer Center, Odessa, USA (6); Dr Bidin, Ospedale Civile, Piacenza, Italy (5); CJH Van De Velde, Academisch Ziekenhuis, Leiden, Netherlands (4); I Alvarez, Hospital Nuestra Sra de Aranzazu, San Sebastian, Spain (4); J Graham, Taunton & Somerset NHS Trust, Taunton, UK (4); J Stone, North Florida Hematology/Oncology Association, Jacksonville, USA (4); M Savin, Texas Oncology, Dallas, USA (4); P Simmonds, Royal South Hants Hospital, Southampton, UK (4); RH Good, Texoma Cancer Center, Sherman, USA (4); C Gaffney, Royal Gwent Hospital, Newport, UK (3); FA Holmes, Texas Oncology, Houston, USA (3); HMA Yosef, Hairmyers Hospital, East Kilbride, UK (3); J Cantrell, Birmingham Hematology and Oncology Assoc, Birmingham, USA (3); J McAleer, Belfast City Hospital, Belfast, UK (3); N Stuart, Gwynedd Hospital, Bangor, UK (3); P Barrett-Lee, Velindre Hospital, Cardiff, UK (3); A Goldhirsch, Instituto Europeo di Oncologia, Milan, Italy (2); A Jones, Royal Free Hospital, London, UK (2); E Mickiewicz, Instituto 'Angel Roffo', Buenos Aires, Argentina (2); J Olivaires, Texas Oncology Physicians Association, Garland, USA (2); M Martin, Hospital Clinico San Carlos, Madrid, Spain (2); M Schottstaedt, Longview Cancer Center, Longview, USA (2); O Contreras Ortiz, University Hospital 'Jose de San Martin', Buenos Aires, Argentina (2); R J Grieve, Walsgrave Hospital, Coventry, UK (2); R Taetle, Hematology oncology Association, Tucson, USA (2); RK Agrawal, Royal Shrewsbury Hospital, Shrewsbury, UK (2); S Akbain, Texas Oncology Physicians Association, Arlington, USA (2); S Della-Fiorentina, Liverpool Hospital, Liverpool, Australia (2); T Cartwright, Ocala Oncology Center, Ocala, USA (2); T Howell, Withington Hospital, Manchester, UK (2); T Perren, St James’ University Hospital, Leeds, UK (2); V Harvey, Auckland Hospital, Auckland, New Zealand (2); A Greenspan, Oncology/Hematology Assoc, Indianapolis, USA (1); A Jones, Whittington Hospital, London, UK (1); A Makris, Luton & Dunstable Hospital, Luton, UK (1); A Nunez de Pierro, Hospital Fernandez, Buenos Aires, Argentina (1); A Patel, St Margaret's Hospital, Epping, UK (1); B Zanger, Memorial Southwest Hospital, Houston, USA (1); J Martinez, Hospital Britanico, Buenos Aires, Argentina (1); JT Fleagle, Rocky Mountain Cancer Center, Boulder, USA (1); L Garbo, Capital District Hematology, Latham, USA (1); M Merlano; D Perroni, Ospedale S Croce, Cuneo, Italy (1); M Modiano, Hematology and Oncology, Tucson, USA (1); MK Croghan, Arizona Oncology Association, Tucson, USA (1); R Labianca, Ospedali Riuniti di Bergamo, Bergamo, Italy (1); R Marschke; DC Medgyesy, Rocky Mountain Cancer Center, Fort Collins, USA (1); R Orti, Hospital Italiano de Buenos Aires, Buenos Aires, Argentina (1); S Salvagni, Azienda Ospedaliera, Parma, Italy (1).

Other significant contributors: Sam Coster and Rosemary Murray: Cancer Research UK Psychosocial Oncology Group, Brighton & Sussex Medical School, University of Sussex, Brighton, UK; Lorna Gibson, Sarah Fancy, Kathryn Wright, Lynda Micklewright, Samiya Chaudhry, and Marie Miller: ICCG Data Centre, Imperial College, London, UK; Steve Canham: Institute of Cancer Research, Sutton, UK; María Alejandra Diaz and Hernan Giancarlo: Argentine Breast Cancer Group, Argentina; Leah Rutkay, Karen Hancock, Dianne Lindsay, Amanda McCue and Rebecca Ramm: ANZ Breast Cancer Trials Group, Australia; Irene Van Hoorebeeck and Patrick Therasse: European Organisation for Research and Treatment of Cancer, Brussels; Rosa Gonzalezgalindo and Jose Ignacio Ubeda: Grupo Espanol de Investigacion del Cancer de Mama, Spain; Roberta Camisa: Gruppo Oncologico Italiano di Ricerca sul Cancro, Italy; Helen Mueller, Anna Saurer and Melanie Strausak: International Breast Cancer Study Group, Switzerland; Marie Emson: International Collaborative Cancer Group, England; Katya Mantoan and Bruno Mitozzi: Italian Trials in Medical Oncology, Italy; David Ryder: North West England, England; Stephanie Pollard: Yorkshire Breast Group, England; Sarah Burns and Lucy Branston: Wales Cancer Trials Network, Wales; Erin Fenske, Candace Branam and Rita Lopez: U.S. Oncology, USA.

**Prevalence of severe cases of individual ES symptoms**

Graphs are grouped in to common (highest percentage reported at any time-point>20%), moderately common (highest percentage reported at any time-point 10-20%), rare (highest percentage reported at any time-point <10%) symptoms

**Common symptoms**

Figure A1: Reporting of poor sleep over time in the QoL study

Key: Solid bars indicate % patients reporting poor sleep on-treatment; Hatched bars indicate % patients reporting poor sleep post-treatment

Figure A2: Reporting of severe hot flushes over time in the QoL study

Key: Solid bars indicate % patients reporting severe hot flushes on-treatment; Hatched bars indicate % patients reporting severe hot flushes post-treatment

Figure A3: Reporting of severe night sweats over time in the QoL study

Key: Solid bars indicate % patients reporting severe night sweats on-treatment; Hatched bars indicate % patients reporting severe night sweats post-treatment

Alternative figure A3: Reporting of severe night sweats over time in the QoL study

Key: Solid bars indicate % patients reporting severe night sweats on-treatment; Hatched bars indicate % patients reporting severe night sweats post-treatment

Figure A4: Reporting of severe lack of energy over time in the QoL study

Key: Solid bars indicate % patients reporting severe lack of energy on-treatment; Hatched bars indicate % patients reporting severe lack of energy post-treatment

Figure A5: Reporting of severe weight gain over time in the QoL study

Key: Solid bars indicate % patients reporting severe weight gain on-treatment; Hatched bars indicate % patients reporting severe weight gain post-treatment

**Moderately common symptoms**

Figure A6: Reporting of severe cold sweats over time in the QoL study

Key: Solid bars indicate % patients reporting severe cold sweats on-treatment; Hatched bars indicate % patients reporting severe cold sweats post-treatment

Figure A7: Reporting of severe nervous feeling over time in the QoL study

Key: Solid bars indicate % patients reporting severe nervous feeling on-treatment; Hatched bars indicate % patients reporting severe nervous feeling post-treatment

Figure A8: Reporting of severe mood swings over time in the QoL study

Key: Solid bars indicate % patients reporting severe mood swings on-treatment; Hatched bars indicate % patients reporting severe mood swings post-treatment

Figure A9: Reporting of severe diarrhoea over time in the QoL study

Key: Solid bars indicate % patients reporting severe diarrhoea on-treatment; Hatched bars indicate % patients reporting severe diarrhoea post-treatment

Figure A10: Reporting of severe bloated feeling over time in the QoL study

Key: Solid bars indicate % patients reporting severe bloated feeling on-treatment; Hatched bars indicate % patients reporting severe bloated feeling post-treatment

Figure A11: Reporting of severe vaginal dryness over time in the QoL study

Key: Solid bars indicate % patients reporting severe vaginal dryness on-treatment; Hatched bars indicate % patients reporting severe vaginal dryness post-treatment

Figure A12: Reporting of severe discomfort with intercourse over time in the QoL study

Key: Solid bars indicate % patients reporting severe discomfort with intercourse on-treatment; Hatched bars indicate % patients reporting severe discomfort with intercourse post-treatment

Figure A13: Reporting of severe breast tenderness over time in the QoL study

Key: Solid bars indicate % patients reporting severe breast tenderness on-treatment; Hatched bars indicate % patients reporting severe breast tenderness post-treatment

**Rare symptoms**

Figure A14: Reporting of severe light-headedness/dizziness over time in the QoL study

Key: Solid bars indicate % patients reporting severe light-headedness/dizziness on-treatment; Hatched bars indicate % patients reporting severe light-headedness/dizziness post-treatment

Figure A15: Reporting of severe headaches over time in the QoL study

Key: Solid bars indicate % patients reporting severe headaches on-treatment; Hatched bars indicate % patients reporting severe headaches post-treatment

Figure A16: Reporting of severe irritable feeling over time in the QoL study

Key: Solid bars indicate % patients reporting severe irritable feeling on-treatment; Hatched bars indicate % patients reporting severe irritable feeling post-treatment

Figure A17: Reporting of severe nausea over time in the QoL study

Key: Solid bars indicate % patients reporting severe nausea on-treatment; Hatched bars indicate % patients reporting severe nausea post-treatment

Figure A18: Reporting of severe vomiting over time in the QoL study

Key: Solid bars indicate % patients reporting severe vomiting on-treatment; Hatched bars indicate % patients reporting severe vomiting post-treatment

Figure A19: Reporting of severe vaginal irritation over time in the QoL study

Key: Solid bars indicate % patients reporting severe vaginal irritation on-treatment; Hatched bars indicate % patients reporting severe vaginal irritation post-treatment

Figure A20: Reporting of severe vaginal bleeding over time in the QoL study

Key: Solid bars indicate % patients reporting severe vaginal bleeding on-treatment; Hatched bars indicate % patients reporting severe vaginal bleeding post-treatment
